# Advances in Single-Cell Multi-Omics and Application in Cardiovascular Research

**DOI:** 10.3389/fcell.2022.883861

**Published:** 2022-06-06

**Authors:** Xingwu Zhang, Hui Qiu, Fengzhi Zhang, Shuangyuan Ding

**Affiliations:** ^1^ Center for Stem Cell Biology and Regenerative Medicine, School of Medicine, Tsinghua University, Beijing, China; ^2^ School of Life Sciences, Tsinghua University, Beijing, China; ^3^ First Hospital of Tsinghua University, Beijing, China

**Keywords:** single-cell technology, multi-omics, epigenomic, integrative analysis, cardiovascular system

## Abstract

With the development of ever more powerful and versatile high-throughput sequencing techniques and innovative ways to capture single cells, mapping the multicellular tissues at the single-cell level is becoming routine practice. However, it is still challenging to depict the epigenetic landscape of a single cell, especially the genome-wide chromatin accessibility, histone modifications, and DNA methylation. We summarize the most recent methodologies to profile these epigenetic marks at the single-cell level. We also discuss the development and advancement of several multi-omics sequencing technologies from individual cells. Advantages and limitations of various methods to compare and integrate datasets obtained from different sources are also included with specific practical notes. Understanding the heart tissue at single-cell resolution and multi-modal levels will help to elucidate the cell types and states involved in physiological and pathological events during heart development and disease. The rich information produced from single-cell multi-omics studies will also promote the research of heart regeneration and precision medicine on heart diseases.

## Introduction

With the development of massively parallel DNA sequencing technologies, reading the whole genome and transcripts in cells has become a promising approach to studying cellular states. Transcriptomes can be amplified to sufficient quantity for high-throughput sequencing. Compared to conventional bulk sequencing methods, single-cell analysis resolves cell heterogeneity otherwise masked by bulk analysis. It enables new cell types and intermediate states to be discovered based on transcriptional and epigenetic signatures of individual cells. These cells are often missed in conventional studies using pre-defined markers or lineage tracing studies due to the lack of known markers or precursors. This is particularly important for studying human primary tissue samples, where knowledge from model organisms may not always be applicable. Recently, single-cell analysis has been used to study cell fate changes during the reprogramming of cardiomyocytes (CMs) (Liu et al., 2017), in the heart from gene knock-out animals (*Nkx2.5* KO, *Hand1* KO, etc.) (DeLaughter et al., 2016; de Soysa et al., 2019) or mouse model of myocardial infarction (MI), heart failure (HF), etc. (Martini et al., 2019; Wang Z. et al., 2020). Researchers could detect early changes in the transcriptome before the phenotype and cell fate changes in all cardiac cells, including CMs, fibroblast cells, immune cells, etc. The single-cell multi-omics atlas offers multi-modal and panoramic views of diseased hearts, allowing scientists to analyze from many different directions. Moreover, cell trajectory analysis based on single-cell data could reveal the continuum of cell fate transition, adding the temporal information missed from samples collected at particular timepoints.

The first single-cell whole-transcriptome profiling was reported by Tang et al., in 2009. Single oocytes were picked by mouth pipette, followed by reverse transcription, polyA tailing, and second-strand synthesis (Tang et al., 2009). Later, various single-cell capture, barcoding, and pooling strategies were developed to handle single cells more efficiently and automatically, which greatly facilitated the development of single-cell multi-omics sequencing.

The cell-type-specific epigenetic landscape is shaped by unique distributions of DNA methylation, chromatin accessibility, key histone marks, and transcription factors (TFs) binding across the genome. Genome-wide profiling of these epigenetic features facilitated the mechanistic study of the developmental process and gene function. The epigenomic profiling technologies include Assay for Transposase Accessible Chromatin with high-throughput sequencing (ATAC-seq) (Buenrostro et al., 2013), Chromatin immunoprecipitation assays with sequencing (ChIP-seq) (Park 2009) and DNA methylome sequencing (DNAme-seq), etc. (Meissner et al., 2005; Laird 2010). They usually require many cells because each cell only has one set of genomic DNA, and thus the number of the template is much less than the transcriptome. However, with the development of more powerful tool enzymes and a better streamlining of the procedure, chromatin analysis at the single-cell level has become a reality. It helps to reveal the heterogeneity in the epigenome and multi-modal regulation of gene expression.

To capture multi-modal information of single cells, samples can be split into multiple parts for multi-omics sequencing separately. However, multi-omics sequence information from the same cells can genuinely reveal the correlation between different layers of epigenetic modification and gene expression (Ma A. et al., 2020). With the explosion of sequencing data from multiple modalities, many algorithms were developed to analyze multi-modal information, compare data generated from different platforms, and remove batch effects for single-cell datasets. Here we will also briefly summarize the bioinformatic methods to merge or compare single-cell datasets and cross-reference epigenomic data derived from single cells with Genome-wide association studies (GWAS).

This review will summarize the platforms for single-cell capture and multi-omics sequencing, the recently emerged technologies and analysis tools, and their potential application in cardiovascular research.

## Development of Single-Cell RNA-Seq Platforms

Several effective methods were developed to amplify the transcriptome of a single cell, including tagging transcripts with T7 promoter and *in vitro* transcription by T7 RNA polymerase (Hashimshony et al., 2012) or template switching by M-MLV reverse transcriptase and template switching oligo (SMART-seq2 (Picelli et al., 2014)). These methods improved the sensitivity and streamlined the procedure of cDNA library generation, paving the way for higher throughput single-cell RNA-seq.

Various platforms are engineered to capture single cells to suit different needs. Based on where the cell barcoding takes place, the single-cell sorting strategy can be classified into three types, cell-per-well (CPW) strategy (Tang et al., 2009), droplet-based strategy (Klein et al., 2015; Macosko et al., 2015; Zheng et al., 2017; Zilionis et al., 2017; Briggs et al., 2018; Farrell et al., 2018; Wagner et al., 2018; Zhang X. et al., 2019), and single-cell combinatorial indexing (SCI) strategy (Cao et al., 2017; Rosenberg et al., 2018).

**FIGURE 1:**
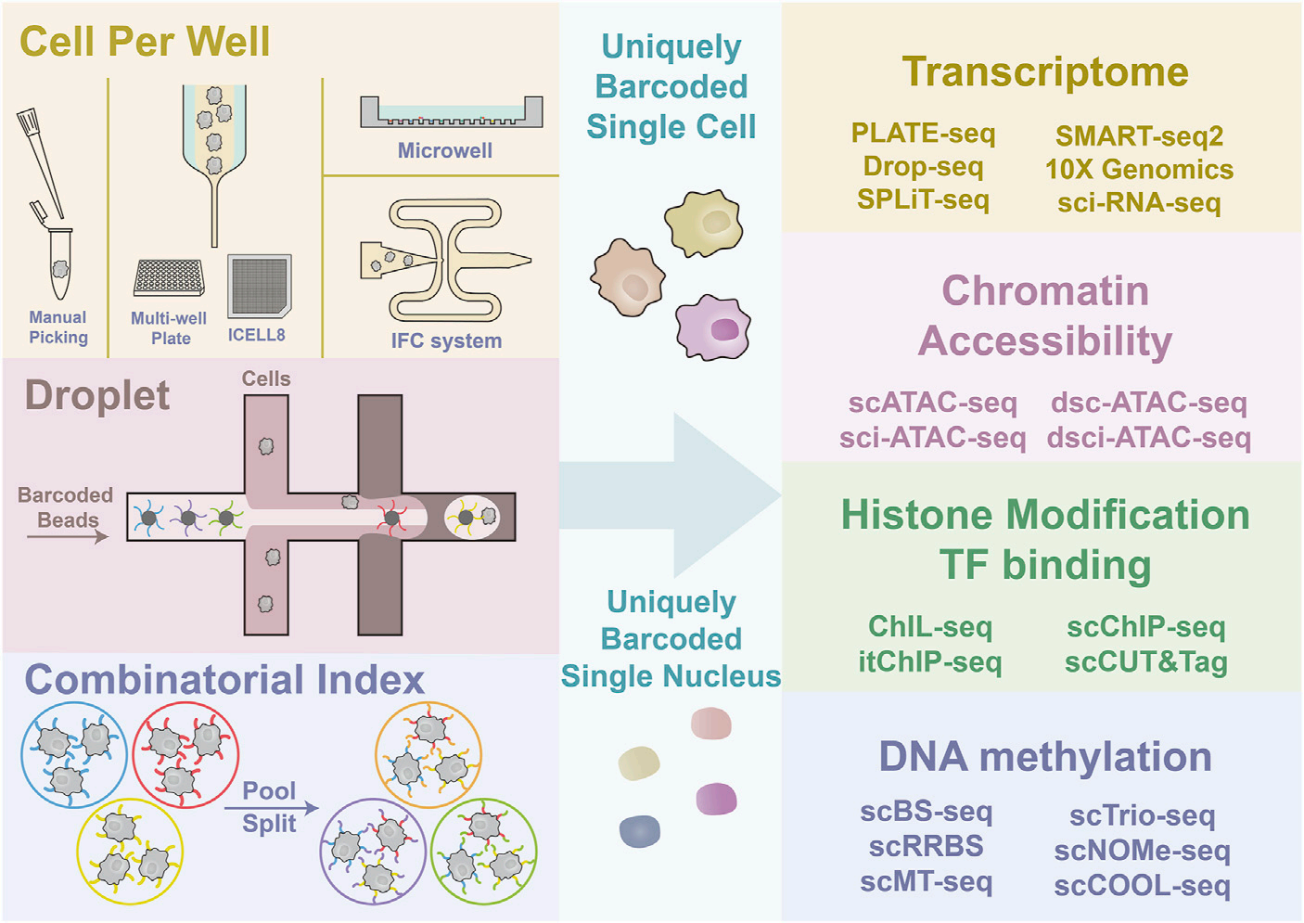
Graphical illustration of single-cell sorting strategy and multi-omics sequencing. The left side illustrates the principles of different cell sorting strategies, CPW, droplet-based, and SCI. Four types of commonly adopted CPW methods include manual picking of single cells; FC and ICELL8, which are both dependent on Hydrodynamic focusing for single-cell flow generation; microwell platform, which uses gravity and size selection for cell capture; IFC system, which integrates multi-steps of library construction. Droplet-based strategies use the co-flow system to generate the emulsion drops containing one cell and one uniquely barcoded bead to barcode the cell by capturing nucleic acid. SCI strategy barcode tens of cells simultaneously and repeat the barcoding after pooling and splitting. Multiple rounds of pooling and splitting followed by barcoding result in a high proportion of uniquely barcoded single cells. The ultimate goal of different cell capture and labeling strategies is to give each cell or nuclei a unique barcode. Then the multi-omics library construction can be performed.

CPW strategies [e.g., manual picking, integrated fluidic circuit (IFC) system, ICELL8 platform, and Microwell-Seq] directly put one cell into one well or tube, followed by library construction separately. Droplet-based strategies [e.g., inDrops, Drop-seq, and 10✕ Genomics Chromium et al. (10✕)] use a “co-flow” device for cell encapsulation with barcoded microparticles to capture mRNA. SCI strategies use the intact cell or nucleus as an indexing unit, and each cell will, with high probability, have a unique barcode combination after multiple rounds of split-pool indexing (Figure 1). Here we will briefly summarize the advantages and features of the three strategies.

### Cell-Per-Well Strategy

The earliest CPW strategy relied on labor-intensive manually picking single cells into each tube (Tang et al., 2009; Islam et al., 2011; Wen and Tang 2016). Later, high-throughput platforms for CPW strategy are developed. IFC system [e.g., Fluidigm C1 (Wu et al., 2014)] and ICELL8 could perform cell isolation, lysis, and reverse transcription in dedicated chambers for each cell in an automated workflow, allowing profiling of several hundred cells per run. Sampling bias should be considered if input cells have different sizes. Technically, the IFC system is more suited for cells less than 25 µm in diameter, while ICELL8 and flow cytometry-based sorting have different nozzle sizes for bigger cells to pass with minimal damage.

Microwell-based methods (agarose-based microwell or BD Rhapsody™ microwell cartridge) offer another solution to improve the throughput and partially overcome the constraints in cell size. (Fan et al., 2015; Han et al., 2018). These methods cast plates containing over 10^5^ microwells. Each microwell can seed one cell and one barcoded bead. Cells are seeded into microwells by gravity, followed by beads settling. After cell lysis in the plate, RNA transcripts are released into the microwell and captured by beads conjugated with barcoded primers; afterward, the beads are collected, and subsequent reverse transcription is performed in bulk. Compared to microfluidic-based workflows, microwell-based workflows offer higher cell throughput, higher tolerance for cell size variation, and gentle treatment by eliminating shear stress from micro-fluidic channels. Microwell-based methods can interrogate a high volume of samples in a single assay, albeit producing lower sequencing depth. It is an excellent choice for large-scale single-cell profiling. For example, Han et al. depicted the cell atlas of mice (more than 400 thousand cells) and human cells (more than 700 thousand cells) using the microwell platform (Han et al., 2018; Han et al., 2020).

### Droplet-Based Strategy

Droplet-based platforms pack single cells and barcoded beads into nanoliter droplets in oil emulsions and utilize these tiny droplets as the reaction chambers. This approach eliminates the need for microfluidic chambers or individual tubes and significantly improves the throughput.

There are three state-of-art droplet-based scRNA-seq strategies, inDrops (Zilionis et al., 2017), Drop-seq (Macosko et al., 2015), and 10✕ genomics. These three systems employ different types of beads (deformable hydrogel beads for inDrop, dissolvable gel beads for 10✕, and hard, brittle resin beads for Drop-seq). All three systems perform cell lysis and mRNA capture in a droplet. However, the reverse transcription reaction is inside droplet for inDrops, out of droplet for 10✕ and Drop-seq. 10✕ and Drop-seq pipelines can be completed within one day. According to a benchmark comparison among the three droplet-based systems, 10✕ shows the highest sensitivity and proportion of effective reads and relatively fewer mismatches in cell barcodes, indicating good quality control in bead fabrication and well-optimized chemistry in DNA synthesis (Zhang X. et al., 2019). However, Drop-seq has considerable advantages in input and cost (∼$0.1 per cell for Drop-seq, ∼$0.5 for 10✕) with a slight compromise in sensitivity. Overall, droplet-based platforms yield very high cell throughput (10^3^∼10^4^ cells per run) with sufficient gene coverage (average 10–50 thousand unique reads for each cell, 3000–6000 detected genes), but they all require specific instruments for droplet generation and have a limitation on cell size (less than 30 µm in diameter for most pipelines).

### Combinatorial Index-Based Strategy

The single-cell combinatorial indexing (SCI) method is a new high-throughput single-cell sequencing paradigm. It takes advantage of the intactness of the cell nucleus during reverse transcription and Tn5 tagmentation. The nuclear membrane is a physical barrier where the indexing reaction occurs inside. Cells are indexed in dozens of wells simultaneously, and multiple rounds of indexing are carried out through pooling and splitting cells into other dozens of wells. After multiple rounds of split-pool indexing, each nucleus will, with high probability, have a unique barcode combination. The SCI method quickly gained popularity because it requires no special instruments other than conventional flow cytometry equipment, and the cost per cell is vastly reduced.

In 2017, Junyue Cao et al. developed sci-RNA-seq that involved two rounds of indexing: the first round during the reverse transcription and the second round during the PCR amplification (Cao et al., 2017). They reported over 20,000–50,000 reads per cell using human and mouse cell lines and profiled over 50,000 *C. elegans* cells with a median of 575 Unique Molecular Identifiers (UMIs). Other methods employed more complex indexing steps to increase the input or sample multiplexing. For example, in 2018, Alexander B. Rosenberg et al. developed SPLiT-seq that introduced four rounds of barcoding (one round during reverse transcription, two rounds by ligation, and the final round through PCR amplification), which yielded over 21 million barcode combinations, vastly decreased the probability of barcode collisions (Rosenberg et al., 2018). In 2020, Sanjay R. Srivatsan et al. first used indexed polyadenylated single-strand DNA to tag each sample, then pooled all samples for downstream three rounds of combinatorial indexing, which allowed them to profile >650,000 cells from more than 5000 independent samples, demonstrating the power of SCI methods in High Throughput Screen studies (Srivatsan et al., 2020). SCI strategy has also been employed to profile chromatin accessibility (Cusanovich et al., 2015), genome sequence (Vitak et al., 2017), 3D genome conformation (Ramani et al., 2017), and DNA methylation (Mulqueen et al., 2018) in an ultra-high throughput manner.

## Development and Advance in Single-Cell Epigenomic Sequencing Technologies

Cell fate determination is a highly regulated process accompanied by changes in the epigenetic landscape, including DNA methylation, open chromatin, and histone modifications. Bulk sequencing of these epigenomic modalities has been well-developed and recently adapted to single-cell studies. Here we will briefly summarize single-cell Assay for Transposase Accessible Chromatin with high-throughput sequencing (scATAC-seq), single-cell Chromatin immunoprecipitation assays with sequencing (scChIP-seq), and single-cell DNA methylome sequencing (scDNAme-seq).

### Single-Cell ATAC-Seq

Open chromatin regions often harbor more transcription factor (TF) binding sites and regulatory regions, which are of great interest. Several enzymes preferably cut the open regions of genome DNA [e.g. deoxyribonuclease I (DNase I), Micrococcal nuclease (MNase), Tn5n transposase] and thus have been used to interrogate chromatin accessibility (Jin et al., 2015) (Schones et al., 2008; Valouev et al., 2011). DNase-based probing combined with either restriction enzyme cleavage (Boyle et al., 2008) or size selection (Hesselberth et al., 2009) produced the first accessible chromatin dataset (Crawford et al., 2006; Sabo et al., 2006; Thurman et al., 2012). Later, Buenrostro et al. used Tn5 transposase to bind, fragment, and tag the accessible genome region with sequencing adaptors and developed ATAC-seq (Buenrostro et al., 2013). ATAC-seq significantly streamlined the processes and increased the sensitivity compared to DNase-seq.

In 2015, scATAC-seq was developed. Researchers isolated 254 GM12878 lymphoblastoid cells and used Tn5 tagmentation to profile the accessible chromatin on the CPW platform. The authors showed that the open chromatin profile from single cells strongly correlated with those obtained by DNase-seq and bulk ATAC-seq generated from tens of thousands or millions of cells. An average of 7.3 × 10^4^ fragments were generated from each cell and mapped to the human genome (Buenrostro et al., 2015). Other CPW-based scATAC-seq were also developed later to analyze cells captured by flow cytometry (Chen et al., 2018a; Chen et al., 2018b), Takara ICELL8 (Mezger et al., 2018), or microwell (Chen et al., 2021).

In 2015, Cusanovich et al. developed sci-ATAC-seq based on the SCI strategy (Cusanovich et al., 2015). The initial version of sci-ATAC-seq analyzed hundreds of cells, and the median reads per cell were only 2.5 × 10^3^. However, the theoretical barcode combination of sci-ATAC-seq is sufficient for tens of thousands of single nuclei. In 2018, Shendure and his colleagues performed sci-ATAC-seq on thousands of cells in one experiment and obtained an average of 13,000 de-duplicated unique reads per cell (Cusanovich et al., 2018a; Cusanovich et al., 2018b). Later in 2020, sci-ATAC-seq was upgraded to sci-ATAC-seq3 by performing three rounds of indexing through ligation instead of one-round Tn5 indexing. After five years of optimization, researchers reduced the collision rate from 11% in original sci-ATAC-seq to lower than 4% in sci-ATAC-seq3 and increased the throughput from thousands of cells to millions of cells. With sci-ATAC-seq3, the authors profiled 1.6 million cells from 59 fetal samples and generated a human fetal cell atlas of chromatin accessibility (Domcke et al., 2020).

Meanwhile, Buenrostro and his colleague developed dsc-ATAC-seq by employing droplet-based single-cell capturing (Lareau et al., 2019). The super-load beads strategy processed 46,000 adult mouse brain cells from 12 experiments and generated 34,000 median unique reads per cell. 10✕ Chromium also launched a standardized commercialized solution for scATAC-seq with a lower multiplet rate (<1%) and high coverage (27.8 × 10^^3^ mean unique reads per cell) (Satpathy et al., 2019). In addition, dsci-ATAC-seq combined droplet-based dsc-ATAC-seq and SCI strategy, adding another round of indexing by split and pool, which increased the throughput by roughly two orders of magnitude. Caleb et al. had adopted dsci-ATAC-seq to profile more than 60,000 human bone marrow cells with around 3,800 unique reads per cell (Lareau et al., 2019).

### Single-Cell ChIP-Seq

Modification of histone tails regulates the structure of chromatin and significantly influences gene expression. Several well-known histone modifications are associated with active transcription, including H3K4me1 [active and primed enhancers (Local et al., 2018)], H3K4me2 [active and primed promoter (Pekowska et al., 2010)], H3K4me3 [active promoter, mainly at regions flanking transcription starting sites (Liu et al., 2016)], H3K27ac [active enhancers and promoters (Creyghton et al., 2010)], H3K36me3 [actively transcribed genes (Musselman et al., 2013)]. Histone modifications associated with transcription repression are also of great importance, especially H3K27me3 [repressed loci (Liu et al., 2016)] and H3K9me3 (marking heterochromatin) (Consortium, Encode Project, 2012; Nicetto and Zaret 2019).

ChIP-seq has been widely adopted to profile different types of histone modification and define various regulatory elements in cardiovascular development and disease studies (Creyghton et al., 2010; Shen et al., 2012; Akerberg et al., 2019; Nakato et al., 2019; Reyes-Palomares et al., 2020). Henikoff and his colleagues developed Cleavage Under Targets & Release Using Nuclease (CUT&RUN), where antibodies of specific histone modifications were bound by protein A which was covalently linked to MNase. The MNase cleavages DNA on either side of the antibody binding site. CUT&RUN combined the endonuclease activity of MNase and the specificity of the antibody. It also bypassed the immunoprecipitation step and significantly simplified the ChIP-seq process. Henikoff group showed that CUT&RUN generated a dataset with a higher signal-to-noise ratio. Rotem et al. adopted CUT&RUN to the droplet-based single-cell capturing platform to profile H3K4me3 and H3K4me2 of mouse embryonic cells, demonstrating that the sensitivity of CUT&RUN is sufficient for single-cell profiling (Rotem et al., 2015). Henikoff group further developed Cleavage Under Targets and Tagmentation (CUT&Tag), where MNase was replaced by Tn5 transposase, which offers the advantage of direct tagmentation of the antibody binding site. CUT&Tag was widely used to profile the genome-wide association of TFs and histone modifications. Although CUT&RUN and CUT&Tag avoided the immunoprecipitation procedure, some researchers still use the term ChIP-seq to nominate these two methods. CUT&Tag has been combined with different single-cell capture strategies to profile histone modification at the single-cell level (e.g., CPW: ChIL-seq (Harada et al., 2019), SCI: itChIP-seq (Ai et al., 2019) and droplet-based strategy: scChIP-seq, scCUT&Tag (Grosselin et al., 2019; Kaya-Okur et al., 2019; Bartosovic et al., 2021)).

### Single-Cell DNA Methylation Sequencing

DNA methylation is a fundamental epigenetic modality closely linked to cell identity and transcriptional regulation. Methylation at the promoter region often marks silenced genes, while methylation in the gene body is a feature of actively transcribed genes (Jones 2012). Bisulfite conversion leads to the deamination of unmethylated cytosines into uracil. Whole-genome bisulfite sequencing (WGBS) can profile methylated CpG sites across the genome at the single-nucleotide resolution but require a large amount of input DNA and deep sequencing. In comparison, reduced representative bisulfite sequencing (RRBS) used the MspI restriction enzyme to enrich CpG sites, thus reducing the sequencing cost at the expense of relatively lower genome coverage.

Based on bulk WGBS and RRBS methods, scRRBS (Guo et al., 2013; Guo et al., 2015) and scBS-seq (Smallwood et al., 2014; Clark et al., 2017) are the first two scDNAme-seq methods. Importantly, scBS-seq is the first method to use the post bisulfite adaptor tagging (PBAT) strategy to minimize the impact of DNA degradation during the bisulfite conversion step. A scDNAme-seq database of more than 8,000 cells across 29 cell types was obtained and provided a valuable database for DNA methylome study (Zong et al., 2022).

Several methods have been developed to profile DNA methylome, transcriptome, and other epigenetic information simultaneously. Most methods physically separate the nucleus and the cytoplasm for bisulfite sequencing and RNA-seq, such as scMT-seq (Hu et al., 2016) and scTrio-seq (Hou et al., 2016). Other methods [scNOMe-seq (Pott 2017) and scCOOL-seq (Li et al., 2018)] utilized GpC methylase, M.CviPI, which specifically methylate GpC sites in the open chromatin regions. This approach enabled profiling DNA methylation and chromatin accessibility simultaneously.

## Sing-Cell Multi-Omics Sequencing Technologies

### Single-Cell Multi-Omics Sequencing Platform

In recent years, it has become feasible to simultaneously profile chromatin accessibility, histone modification, TF binding, DNA methylation, and transcriptome from the same cell, namely single-cell multi-omics sequencing (scMulti-omics-seq). ScMulti-omics-seq avoids the need to match datasets from parallel samples, reducing the technical variations. ScMulti-omics-seq can potentially be used to distinguish fluctuation in gene expression and different cell states due to a typical larger feature set than scRNA-seq, which is limited by the number of genes expressed and sequenced (Corces et al., 2016; Chen H. et al., 2019).

The first single-cell RNA-ATAC co-assay method is sciCAR-seq, published in 2018 by Jay Shendure and his colleagues, who had adopted the concept of SCI to scMulti-omics-seq by performing reverse transcription and tagmentation consecutively in the same nuclei before the second round of indexing (Cao et al., 2018). This research provided the proof-of-concept for sciMulti-omic-seq; however, low sensitivity led to a relatively imperfect correlation between gene expression and chromatin accessibility. Paired-seq (Zhu et al., 2019) and SHARE-seq (Ma S. et al., 2020) significantly upgraded the throughput by multiple rounds of indexing. In addition, tagmentation of the genome before reverse transcription prevented cDNA contamination in ATAC libraries due to the potential tagmentation of RNA/DNA hybrids (Di et al., 2020; Lu et al., 2020). Another strategy for transcriptomic chromatin accessibility co-assay is to use splint oligo to bridge tagmented DNA and oligo (dT) bearing barcoded beads so that they can be barcoded with captured RNA and sequenced together (Chen S. et al., 2019). Other studies try to segregate cytosol and nucleus from the same cell and perform scRNA-seq together with scATAC-seq (Liu L. et al., 2019) or scDNAme-seq based on either scBS-seq (Angermueller et al., 2016) or RRBS method (Hu et al., 2016).

Recently published Paired-tag (Zhu et al., 2021) and CoTECH (Xiong et al., 2021) have accomplished the co-assay of single histone modification and transcriptome by performing CUT&Tag and reverse transcription sequentially utilizing the SCI strategy. CoTECH adopted one round of split-pool, leading to relatively lower throughput (theoretical barcode complexity is about 10^4^). Paired-tag adopted three rounds of barcoding through ligation, reaching a theoretically thousand folds higher complexity. Unique reads generated by Paired-tag varied among different histone markers but generally fell within 1-10k, sufficient for analyzing differentially modified regulatory elements. CoTECH generates a dataset with a slightly lower number of unique reads. Zhu et al. profiled ∼70,000 mouse brain cells on H3K4me1, H3K4me3, H3K27ac, H3K27me3, and H3K9me3 genome-wide association and the transcriptome. They successfully partitioned cells into different neuron types using H3K4me1 and H3K27ac modals alone, but H3K4me3, H3K27me3, and H3K9me3 modals failed to resolve the cellular heterogeneity and only clustered cells into broad brain cell types, indicating that it may be unfeasible to link different histone modification modalities directly if matched RNA information is not profiled. Using the transcriptome to bridge with other modalities, they assembled five histone modification datasets to form a comprehensive epigenetic landscape of mouse neurons.

Paired-tag and CoTECH can only profile transcriptome and one single histone modification at a time, so it is not possible to study the relationship between different histone modifications around the same genomic region. To map multiple histone modifications simultaneously in the same cell, multi-CUT&Tag (Gopalan et al., 2021) and MulTI-Tag (Meers et al., 2021) are developed. Both used indexed pA-Tn5 to connect with antibodies of different histone modifications. Multi-CUT&Tag used pre-assembled indexed pA-Tn5 and antibodies for different histone modifications, while MulTI-Tag adopted a covalently conjugated Tn5-antibody complex. According to the preliminary result from Gopalan et al., covalent conjugation significantly removed the cross-contamination between different histone modification maps. Interestingly, MulTI-Tag reported that about 10% of reads would have a different index in each end, which indicated that this region is co-occupied by two histone modifications in the same cell. However, the mix-indexed ratio reported by multi-CUT&Tag is higher (18–20%), possibly due to cross-contamination of histone modalities. Generally, Multi-CUT&TAG and MulTI-Tag are promising tools to investigate the relationship between different histone modifications and their influence on the transcriptome.

### Integrative Analysis of Multiple Modalities

New bioinformatics methods are needed to analyze the vast amount of data generated from scMulti-omics sequencing experiments. Comparative analysis of the same modality, e.g., scATAC-seq, from different experiments turns out to be challenging, as researchers often need to remove batch effects due to different cell capturing strategies, library construction methods, or sequencing platforms. The choice of batch-removal strategy can be significantly determined by the research topic. According to a benchmark study (Luecken et al., 2022), if cell identities are known, that is, one is not expecting novel cell types, then it should be beneficial to integrate scRNA-seq batches via scANVI (Xu et al., 2021) or scGen(Lotfollahi et al., 2019). However, Scanorama (Hie et al., 2019) and scVI (Lopez et al., 2018) are recommended for the large unlabeled dataset, while Harmony (Korsunsky et al., 2019) seems more beneficial for the unlabeled smaller dataset with distinct biological signal (Tran et al., 2020).

Multi-omics data analysis needs to consider distinct modalities’ different sizes and unique nature. Chromatin accessibility and DNA methylome datasets contained information across the whole genome, and histone modifications also mark a significant fraction of the genome. These datasets are significantly larger than the transcriptome (usually no more than 5% of the genome) and TF binding regions. Dozens of computational strategies have been developed to perform integrative analysis of multi-modal datasets, including matched (multi-omics data from the same cell) or unmatched datasets (different modalities profiled from different cells) (Miao et al., 2021). For matched datasets, scAI (Jin et al., 2020), and MOFA+ (Argelaguet et al., 2020) could integrate scRNA and scATAC data. There are more algorithms developed to integrate unmatched scATAC-seq and scRNA-seq. MAESTRO (Wang C. et al., 2020) performs well on a dataset after manually annotating cell types and then analyzing the clustered cell-type level. LIGER (Welch et al., 2019) and Seurat 3.0 (Stuart et al., 2019) integrate scATAC-seq and scRNA-seq data by first predicting putative gene expression by nearest accessibility peak and integrating predicted and observed expression data. SCOT (Demetci et al., 2022) constructed similarity matrixes for each modality separately and integrated them through an optimal transport algorithm. LIGER also performs well for integrative analysis of transcriptome and DNA methylome data but needs a converted feature set, while MMD-MA (Liu J. et al., 2019), UnionCom (Cao et al., 2020) do not require feature matching.

GWAS has identified hundreds of thousands of genetic variants associated with the broad spectrum of human traits and diseases, most of which are noncoding (Claussnitzer et al., 2020). It is challenging but informative to link the noncoding GWAS variants to specific biological processes, pathways, or putative target genes. VAMPIRE has tried to cross-reference datasets of different modalities (e.g., epigenome, transcriptome, and 3D genome conformation) to annotate GWAS loci (Sun et al., 2022). scATAC-seq and scChIP-seq can also bridge noncoding GWAS variants with specific cell types, which provides valuable insights into the etiology and pathology of diseases (Ord et al., 2021). In addition, Cicero could predict cis-regulatory DNA interactions and construct chromatin co-accessibility networks, then correlated GWAS loci to specific genes within the network (Pliner et al., 2018).

## Application of Multi-Modal Single-Cell Sequencing in Cardiovascular Research

### Application of scRNA-Seq to Study the Development of the Cardiovascular System

In recent years, increasing numbers of studies employed scRNA-seq to depict the cell atlas of embryonic, adult, and diseased heart and vasculatures, such as dilated cardiomyopathy (See et al., 2017; Nomura et al., 2018; Rao et al., 2021), congenital heart defect (Suryawanshi et al., 2020), hypoplastic left heart syndrome (Miao et al., 2020), aortic stenosis (Nicin et al., 2020; Nicin et al., 2022), heart failure (Nicin et al., 2020), calcific aortic valve disease (Xu et al., 2020), Ascending thoracic aortic aneurysm (Li et al., 2020), and ischemic cardiomyopathy (Rao et al., 2021) (Table 1).

**TABLE 1:** Research adopting scRNA-seq for *in vivo* human heart.

Year	Topic	Organ/Tissue	Sequencing	Trait/Disease	Strategy	Throughput	Main findings/Contribution to the field	Analyzing methods
2017	Single cardiomyocyte nuclear transcriptomes reveal a lincRNA-regulated de-differentiation and cell cycle stress-response *in vivo*	Adult heart (LV)	scRNA-seq	End-stage dilated cardiomyopathy (DCM)	Fluidigm C1	116 nuclei	Sub-populations of cardiomyocytes displays upregulation of cell cycle, and de-differentiation genes during the endogenous myocardial stress response; Nodal lincRNAs act as key regulators of CM cell cycle during myocardial stress response	WGCNA for gene module detection; Quadrant analysis for cell heterogeneity analysis; Coding Potential Assessment Tool (CPAT) for LncRNA analysis
2018	Cardiomyocyte gene programs encoding morphological and functional signatures in cardiac hypertrophy and failure	Adult heart	scRNA-seq	Dilated cardiomyopathy (DCM)	manually picked cell follow by SMART-seq2	10 DCM (340 cells) 1 Healthy (71 cells)	Trajectory of CM remodeling in repsons to pathological stimuli; Gene modules for CM hypertrophy and filure; Molecular and morphological dynamics of CM leading to heart failure	Random Forest for gene module detection; Weighted gene co-expression network analysis (WGCNA) for gene module detection; Pseudo-time analysis for trajectory modeling
2019	A Spatiotemporal Organ-Wide Gene Expression and Cell Atlas of the Developing Human Heart	embryonic heart (4.5–5PCW, 6.5–7PCW, 9PCW)	spatial RNA-seq scRNA-seq	Healthy	*in situ* RNA-seq 10×	3,115 spots 3,717 cells	Spatiotemporal gene expression of human heart development at single-cell resolution; Distribution, spatial organizaiton, and roles of diverse cell types in embryonic heart	pciSeq for creating probilistic spatial cell map
2019	Single-Cell Transcriptome Analysis Maps the Developmental Track of the Human Heart	embryonic/fetal heart (5PCW–25PCW)	scRNA-seq	Healthy	modified STRT-seq	4,948 cells	Transcriptonal profiling of human heart at single-cell level from early to late developmental stage	Pseudo-time analysis for trajectory modeling; Gene set enrichment analysis (GSEA) and Kyoto Encyclopedia of Genes and Genomes (KEGG) for signaling pathway enrichment
2020	Cell atlas of the foetal human heart and implications for autoimmune-mediated congenital heart block	fetal heart (19–21PCW)	scRNA-seq	Congenital heart block (CHB)	10×	3 Healthy (12,461 cells) 1 CHB (5,286 cells)	Several uncharacterized cell subpopulations are identified; CHB heart shows diversity in interferon-stimulated gene expression across cell types and increased matrisome expression in stromal cells	TF enrichment analysis; Interferon response score calculation for CHB characterization; Matrisome enrichment analysis
2020	Intrinsic Endocardial Defects Contribute to Hypoplastic Left Heart Syndrome	fetal heart ventricular free wall (12 PCW)	scRNA-seq	Hypoplastic left heart syndrome (HLHS)	10×	4,523 CD144+ cells 5,477 CD144- cells	Endocardial defect in HLHS lead to impaired endocardial to mesenchymal transition and angiogenesis, as well as reduced proliferation and maturation of CM by disrupting fibronectin-integrin signaling	Receptor-ligand analysis
2020	Single-cell reconstruction of the adult human heart during heart failure and recovery reveals the cellular landscape underlying cardiac function	Adult heart (LV, LA)	scRNA-seq	Heart failure (HF)	ICELL8-scRNA-seq	14 Healthy (12,266 cells) 6 HF (5,933 cells)	Inter- and intracompartemental CM heterogeneity; Compartment-specific NCM works as major cell-communication hubs Cellular composition and interaction networks of the adult human heart from normal to disease state	Pseudo-time analysis for trajectory modeling; Regulon analysis for regulatory network activity accessment; Receptor-ligand analysis; Cell similarity calculation
2020	Cell-Type Transcriptome Atlas of Human Aortic Valves Reveal Cell Heterogeneity and Endothelial to Mesenchymal Transition Involved in Calcific Aortic Valve Disease	Adult heart (aortic valve leaflets)	scRNA-seq	Calcific aortic valve disease (CAVD)	10×	4 CAVD (31,043 cells) 2 Healthy (3,589 cells)	Endothelial to mesenchymal transition of vascular EC plays important roles in thickening of calcified aortic valve leaflets	Pseudo-time analysis for trajectory modeling; KEGG for signaling pathway enrichment
2020	Single-Cell Transcriptome Analysis Reveals Dynamic Cell Populations and Differential Gene Expression Patterns in Control and Aneurysmal Human Aortic Tissue	Adult heart (ascending aorta)	scRNA-seq	Ascending thoracic aortic aneurysm (ATAA)	10×	8 ATAA 3 Healthy (total 48,128 cells)	A comprehensive evaluation of the expression landscape of ascending aortic wall revealed that ERG played an important role in maintaining aortic wall function	Cell-cell junction score and cell-ECM junction score; Cell cycle analysis for cell proliferation state accessment
2020	Transcriptional and Cellular Diversity of the Human Heart	Adult heart (RA, RV, LA, LV)	snRNA-seq	Healthy	10×	287,269 nuclei	A Large snRNA-seq dataset of healthy human heart from different chamber and sex; Chamber-, laterality- and sex-specific transcriptional programs were identified; Specific cell types were linked to common and rare genetic variants of CVD	CellBender for background removal; scVI model for subgroup detection; eQTL mapping to detect disease-associated cell types
2020	Cells of the adult human heart	Adult heart (RA, RV, LA, LV, Septum, Apex)	scRNA-seq snRNA-seq	Healthy	10×	45,870 unsorted cells 78,023 CD45+cells 363,213 nuclei	The research defined the cellular and molecular signatures of the adult healthy heart, and functional plasticity in response to varying physiological conditions and diseases	Deep variational autoencoder for batch alignment; Cell-cell interaction analysis; RNA velocity analysis for cell state evaluation
2021	Resolving the intertwining of inflammation and fibrosis in human heart failure at single-cell level	Adult heart (LV,RV)	scRNA-seq scTCR-seq	Ischemic cardiomyopathy (ICM) Dilated cardiomyopathy (DCM)	10×	3 DCM 3 ICM 2 Healthy (total 165,999 cells)	AEBP1 is a noval crucial cardiac fibrosis regulator in ACTA2+ myofibroblst; CXCL8+CCR2+HLA-DR + macrophages in fibrotic area interact with activated EC via DARC, which potentially facilitate leukocyte recruitment and infiltration in human heart failure	RNA velocity analysis; Cell-cell interaction analysis; Psudo-time analysis for trajectory modeling; TCR analysis for immune cell; Regulatory analysis for TF-target interactions
2021	Single-Cell Transcriptomic Atlas of Different Human Cardiac Arteries Identifies Cell Types Associated With Vascular Physiology	Adult heart (aorta, pulmonary artery, coronary artery)	scRNA-seq	Healthy	10×	3 aortas 2 pulmonary arteries 9 coronary arteries (total 125,253 cells)	An atlas of human nondiseased cardiac arteries and cell heterogenity analysis	pySCENIC for TF inference and AUCell for regulon activity analysis; Psudo-time analysis for trajectory modeling; CCInx for intercellular communication analysis
2021	Cardiac cell type–specific gene regulatory programs and disease risk association	Adult heart (RA, RV, LA, LV)	scRNA-seq snATAC-seq	Healthy	10×	35,936 nuclei	A cell type–resolved atlas of cCREs in human hearts; Chamber-specific differences in chromatin accessibility between ventricles and atria as well as left and right atria	SnapATAC for scATAC data dimensionality reduction MACS2 for identification of accessible chromatin sites Cicero for coaccessibility analysis edgeR for the identification of cell type-specific CRE GWAS variant enrichment analysis
2022	A human cell atlas of the pressure-induced hypertrophic heart	Adult heart (interventricular septum)	snRNA-seq	Cardiac hypertrophy caused by aortic valve stenosis	10×	88,536 nuclei	EFNB2 inhibition, which is expressed by EC, inhuced CM hypertrophy *in vivo* and *in vitro*	Harmony for batch align; Cell-cell interaction analysis; Receptor-ligand analysis

Sampling on cardiovascular tissues, especially from the heart, requires special cautions. The size of CM (100–150 μm by 20–35 μm) poses a significant challenge for single-cell analysis. Cui et al. adopted modified STRT-seq (Li et al., 2017; Cui et al., 2019) to construct single-cell transcriptome libraries, where mouth pipetting was adopted to capture single cells. ICELL8 platform offers an automated solution to harvest giant cells such as CMs. Another widely adopted solution is to construct libraries on single-nucleus and single-cell simultaneously for heart profiling, and the transcriptome data of CM are primarily derived from snRNA-seq (Litvinukova et al., 2020; Tucker et al., 2020). Several other features of the human heart (i.e., compactness, compartmentalization, and heterogeneity) make it even harder to isolate all types of cells unbiasedly with minimal damage. Li Wang et al. adopted both CM-enriched digestion (Guo et al., 2018) and conventional enzyme digestion of the left ventricle and left auricle/left atrial appendage to harvest large quantities of CMs and non-cardiomyocytes (NCMs) and performed single-cell RNA-seq through ICELL8 platform.

Some studies have adopted scRNA-seq to profile human pluripotent stem cell (hPSC) derived CMs. Data mining in the scRNA-seq helped optimize hPSC differentiation protocols (Zhang H. et al., 2019). Yang et al. employed RNA velocity and SLICER-based trajectory reconstruction to identify the critical fate decision process during cardiac reprogramming. They also developed a cell fate index (CFI) algorithm to assess reprogramming progression and provide valuable insights into how to optimize the differentiation methods (Zhou et al., 2019). Based on hPSC-derived CMs, scRNA-seq was also used to measure the gene regulatory network in drug screening (Ballan et al., 2020), cellular processes under physiological (Cyganek et al., 2018; Gambardella et al., 2019; Schmid et al., 2021; Esfandyari et al., 2022) and pathological conditions (Guo et al., 2019) (Kathiriya et al., 2021) and developmental progression (Hulin et al., 2019; Ruan et al., 2019; Sahara et al., 2019).

### Application of Epigenomic Sequencing in the Cardiovascular System

ScATAC-seq, combined with scRNA-seq, provides valuable insights into characteristics underlying developmental plasticity. Information from single-cell chromatin accessibility during the cardiovascular system development can predict the cell-type-specific regulatory TFs through motif enrichment analysis. Furthermore, researchers are more convinced by the more stable epigenetic landscape of the cell for cell-type identification, while RNA expression can be variable and change rapidly (Depuydt et al., 2020; Alexanian et al., 2021; Hocker et al., 2021).

Only a few published research used scATAC-seq to profile embryonic or adult human hearts. Most of them focus on constructing the regulome atlas (Domcke et al., 2020; Zhang et al., 2021). Hocker et al. performed sci-ATAC-seq of around 80,000 cells from adult human hearts without known cardiovascular disease (CVD) and constructed a human cardiac cis-regulatory element (CRE) atlas of different cell types. Apart from CMs, four types of NCMs (cardiac fibroblasts, endothelial cells, smooth muscle cells, and macrophages) were profiled with specific cardiac chamber annotated. Thus, this research depicted a chromatin accessibility map at single-cell resolution with spatial information. scRNA-seq were performed simultaneously, aiding cell type and subtype annotation. In addition, transcriptome profiling also helped to verify and refine the conclusions derived from scATAC-seq. Enrichment of binding motif for the macrophage TF SPI1/PU.1 combined with high SPI1 expression in macrophage helps to define SP1 as the specific TF in cardiac macrophage. The binding motifs of GATA-family TF were enriched in open chromatin regions of EC, CM, and cardiac fibroblast. GATA6 is highly expressed in cardiac fibroblast, while GATA4 and GATA6 in CM and GATA2 in EC) (Hocker et al., 2021). Such combined analysis helps identify active TFs in the development of different lineages.

The human cardiac CRE atlas derived from the scATAC-seq can be a valuable reference to advance our understanding of gene regulatory mechanisms. For example, for cardiac fibroblasts, there is more differential accessible CREs between right and left ventricles than between atriums and ventricles. Researchers have detected several fibroblast-specific CREs at *FN1* gene and adjacent to *MMP2* and *FBLN2* genes, which all showed higher accessibility in the left atrium. These findings may indicate a more activated fibroblast state associated with higher ECM production in the left atrium (Hocker et al., 2021).

## Summary and Future Perspectives

The technology to acquire and analyze multi-omics information from a single cell is rapidly evolving. Single-cell analysis has already provided an unprecedented amount of information about the cellular composition of the heart during development, homeostasis, and diseased conditions. However, there are many pending questions. How does the epigenetic landscape change in different parts of the heart and cell types during embryo development? How do different cardiac cells interact with immune cells during myocardial infarction and heart failure? Are there intermediate cell states during heart regeneration? How do drugs affect different types of cells in the heart? Answering these complex questions requires full resolution of cardiac cell heterogeneity at multiple layers. The multi-modal information at the single-cell level will significantly improve our effort to understand the molecular mechanism regulating cell fate and states in healthy and diseased conditions and find better biomarkers and drug targets to treat cardiovascular diseases.
